# Differences in the correlation between microbiological air sampling and particle counting in operating theatres and hospital cleanrooms

**DOI:** 10.1186/1753-6561-5-S6-P303

**Published:** 2011-06-29

**Authors:** N Hoelscher, AW Friedrich

**Affiliations:** 1 Pharmacy, University Hospital Muenster, Muenster, Germany; 2 Medical Microbiology and Infection Control, University Hospital Groningen, Groningen, Netherlands

## Introduction / objectives

The process of environmental monitoring in cleanrooms of clinical pharmacy and operating theatres is difficult, the measurement of bio burden is time consuming and the result is available several days after the operation. This study examines the correlation between the number of airborne bacteria and particles in operating theatres and cytostatic compounding cleanrooms in order to evaluate if applying solely particle monitoring is sufficient for hygienic-microbiological risk assessment.

## Methods

Over six months 570 air bacteria and particle counts were collected simultaneously at various times during the operation and production of cytostatic infusion with the TSI Optical Particle Counter in three particle sizes (0.5µm, 1µm and 5μm) and a slit type air sampler with five different agars.

## Results

No association between airborne bacteria and particles p-value >0.05 was found in operating theatres (r=0,203) and cleanroom B (r=0,157). In contrast, a high correlation between airborne bacteria and particles was found in cleanroom class C (r=0,758). The correlation increased with increasing number of particle counts (>5000/mÂ³). In cleanroom class A no airborne bacteria count.

## Conclusion

The results of the present study indicate that particle monitoring can not replace microbiological monitoring. The correlation in class C is without relevance for the cytostatic preparation, since in this cleanroom no processing of critical infusion takes place.The current method of combining particle measurements with microbiological monitoring is at present the only efficient method for hygienic-microbiological risk assessment.

## Disclosure of interest

None declared.

